# Medicinal Garden

**Published:** 1859-12

**Authors:** 


### Medicinal Garden.

TO THE LOVERS OF SCIENCE.

In connection with the Atlanta Medical College, and upon the grounds surrounding the building, is in process of establishment, a Garden of Medicinal plants. It is intended to contain all the exotic as well as indigenous plants that can be obtained. From the scarcity of such collections in any of the Gardens to which we have applied, we find it necessary to obtain most of them from the place of their nativity. It is only such as are ornamental that we have been able to procure from Horticulturists. With the assistance, however, of our medical brethren in various parts of the country, we hope soon to have most of those found in the Southern States of the Union. To those physicians, particularly, who have a taste for examining and studying vegetable remedial agents, strewn with such profusion throughout the land, we look for aid in this undertaking. We have already obtained from Gardens and by individual donation over one hundred articles, which have been classified according to their medicinal properties, and planted in their proper places. We will be pleased to exhibit to those who may favor us with such plants and seeds as may fall in their way, their presents in a flourishing condition, designated in a manner respectful to the donor.

This plan of increasing the number of our plants has been suggested by a lover of science and eminent physician of our State, who considers such a collection very desirable, inasmuch as no Garden of the kind can be found in connection with any Medical College in the country. Such as are disposed to aid in this enterprise will please forward, by express or by mail, such articles as they may find it convenient to send, directed to Dr. J. G. Westmoreland, Atlanta, Ga.

